# Atypical glucose response patterns in pregnant women and the association with hypertensive disorders of pregnancy: A retrospective analysis

**DOI:** 10.1097/MD.0000000000044778

**Published:** 2025-09-26

**Authors:** Min Guo, Kaiqi Wu, Huiqing Yang, Binbin Yin, Jinghua Zhang, Ya Xi, Yongying Bai

**Affiliations:** a Department of Clinical Laboratory, Women’s Hospital, Zhejiang University School of Medicine, Hangzhou, China; b Department of Pharmacy, Hangzhou TCM Hospital Affiliated to Zhejiang Chinese Medical University, Hangzhou, China; c Department of Central Laboratory, Children’s Hospital, Zhejiang University School of Medicine, National Clinical Research Center for Child Health, Hangzhou, China.

**Keywords:** glucose response curve, hypertensive disorders of pregnancy, incessant increase, monophasic, oral glucose tolerance test

## Abstract

The oral glucose tolerance test (OGTT) is crucial for diagnosing gestational diabetes mellitus (GDM), yet traditional screening overlooks OGTT curve shapes. Hypertensive disorders of pregnancy (HDP) are common and hyperglycemia is a risk factor, but the relationship between glucose response patterns during OGTT and HDP, remains underexplored. A retrospective cohort study was conducted on 26,084 pregnant women undergoing 75-gram OGTT at 24 to 28 weeks’ gestation. Glucose curves were classified as incessant increase (IIn, continuous increase at 0, 1, and 2 hours) or monophasic (MPh, peak at 1 hour followed by decrease). Multivariable logistic regression evaluated crude and adjusted relative risks (RRs) of HDP, adjusting for maternal age, preconception body mass index (BMI), parity, in vitro fertilization (IVF), gestational weight gain (GWG), birth weight, gestational age, and GDM status. Subgroup analyses stratified by age categories, BMI categories, and GDM status were performed. The IIn pattern was observed in 14.46% of participants. Compared with the MPh group, the IIn group demonstrated a significantly lower crude RR of HDP [0.70, 95% confidence interval (CI): 0.59–0.84, *P* < .001], which persisted after adjustment for confounding variables (adjusted RR = 0.74, 95% CI: 0.61–0.89, *P* < .01). Stratified analysis demonstrated consistent protective effects of IIn curves across maternal age subgroups, as well as among women with and without GDM. The IIn glucose response curve is independently associated with reduced HDP risk, suggesting its potential as a biomarker for personalized risk assessment.

## 1. Introduction

Currently, the oral glucose tolerance test (OGTT) serves as the gold standard for diagnosing gestational diabetes mellitus (GDM) and evaluating glucose metabolism in Chinese pregnant women.^[1]^ Its importance lies in preventing or alleviating significant short- and long-term complications for both mothers and fetuses.^[2–5]^ A typical OGTT response is characterized by a low fasting glucose level, followed by a peak 30 to 60 minutes after glucose ingestion and subsequent decline, which is defined as the monophasic (MPh) curve.^[6]^ During pregnancy, atypical plasma glucose concentrations are occasionally detected during OGTTs. In such instances, the blood glucose curves of pregnant women show a continuous upward trend without a subsequent decrease, referred to as the incessant increase (IIn) curve. Among these patterns, the MPh curve is the most common, while the IIn pattern is relatively rare.^[7,8]^ The clinical significance of this phenomenon during pregnancy has not been thoroughly investigated in the existing literature. Consequently, most obstetricians do not adjust the antepartum management for women with a continuously increasing OGTT curve.

Hypertensive disorders of pregnancy (HDP), encompassing gestational hypertension, preeclampsia, and eclampsia, affect approximately 15% of pregnancies worldwide and account for 14% of maternal deaths.^[9]^ Early identification of high-risk individuals is therefore of paramount importance for improving pregnancy outcomes.^[10]^ Insulin resistance (IR) is a state where normal concentrations of insulin fail to elicit an adequate response from target cells, triggering excessive insulin secretion through negative feedback mechanisms. To maintain stable blood glucose levels, pancreatic β-cells subsequently enhance insulin production, resulting in hyperinsulinemia.^[11]^ Mainstream perspectives suggest that physiological IR facilitates increased glucose supply to the fetus, promoting its growth and development. However, overactivation of IR during pregnancy can lead to reduced nitric oxide levels, dysregulated lipid metabolism, impaired prostaglandin E2 synthesis, and vascular endothelial damage, ultimately contributing to maternal hypertension.^[12]^ Individuals with elevated plasma insulin concentrations and IR often exhibit higher blood pressure compared to those with normal insulin levels.^[13]^

Recent studies have indicated that glucose response patterns during OGTT reflect β-cell function and insulin sensitivity^[14–20]^ which are closely linked to the pathogenesis of HDP. Additionally, insulin secretion levels and function vary across different curve patterns.^[7]^ Thus, investigating potential differences between distinct glucose curve patterns and HDP represents a compelling area of research. Although β-cell function and insulin sensitivity have been recognized as risk factors for HDP, conventional OGTT primarily emphasizes glycemic thresholds, neglecting the significance of glucose response patterns. Nevertheless, the relationship between glucose response patterns and HDP in the Chinese population remains underexplored. This study aimed to explore whether insulin curves are associated with HDP.

## 2. Methods

### 2.1. Study design and subjects

**Figure 1.**
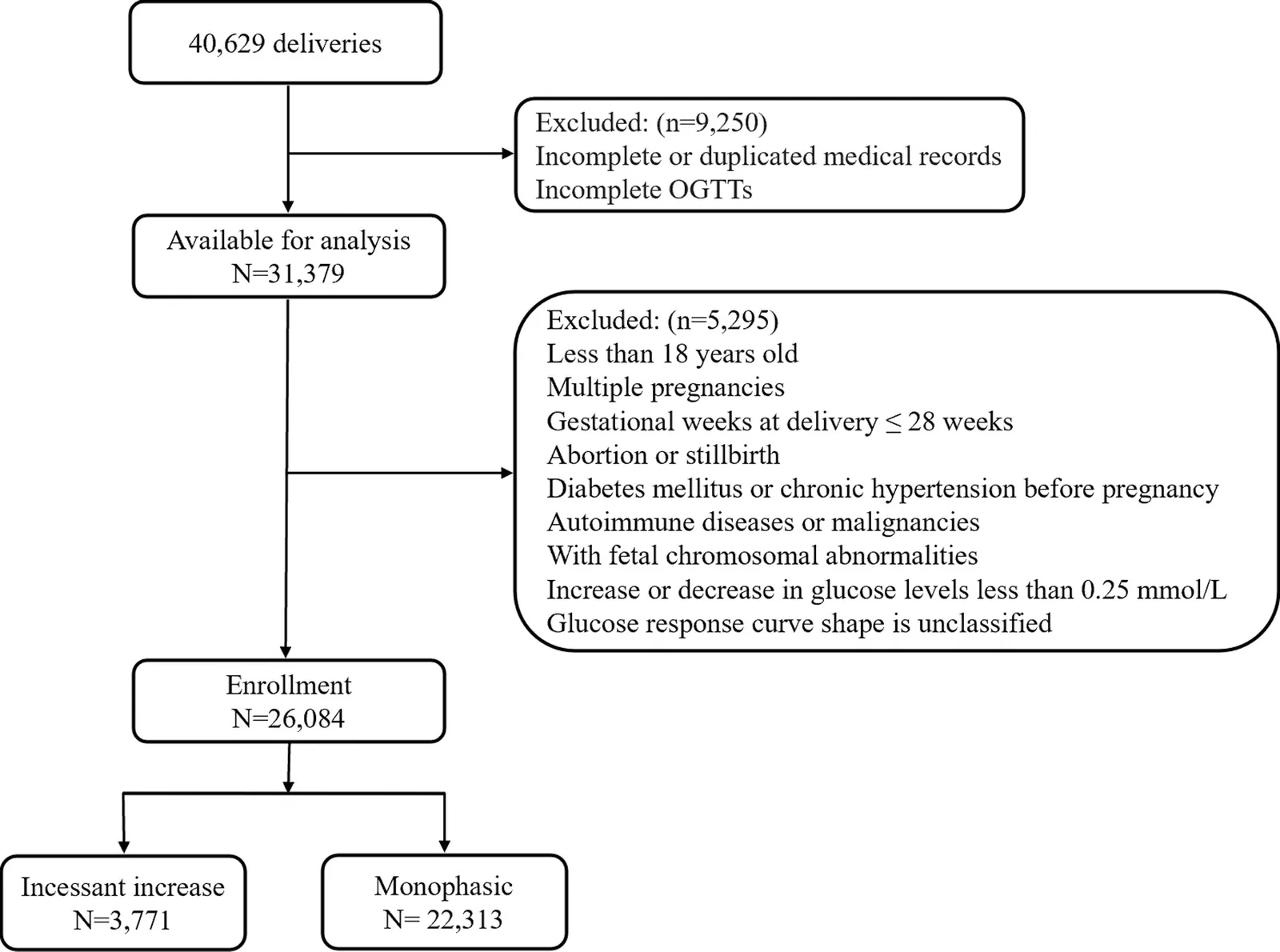
Flowchart illustrating the process of participant enrollment and group assignment. OGTT = oral glucose tolerance test.

This retrospective cohort study included 26,084 women delivering at Women’s Hospital, Zhejiang University School of Medicine, between 2018 to 2019. Ethical approval was obtained from the Institutional Review Board (approval number: IRB-20240021-R; approval date: January 22, 2024), and an informed consent exemption was granted due to the use of anonymized patient records. Women were excluded if they met any of the following criteria: incomplete or duplicated medical records; incomplete OGTT results; under 18 years of age; multiple pregnancies; gestational weeks at delivery ≤ 28 weeks; miscarriage or stillbirth; preexisting diabetes mellitus or chronic hypertension; autoimmune diseases or malignancies; fetal chromosomal abnormalities; or OGTT results with glucose level changes of <0.25 mmol/L. Additionally, participants with unclassifiable OGTT curve shapes were excluded. The screening process for eligible participants is illustrated in Figure 1.

### 2.2. Diagnostic criteria

IIn curves were defined as having continuous glucose increases of ≥0.25 mmol/L at the 0-hour, 1-hour, and 2-hour time points. MPh curves required a peak at 1 hour with ≥0.25 mmol/L increase followed by ≥0.25 mmol/L decrease. A glucose threshold of 0.25 mmol/L was adopted to minimize fluctuations caused by analytical variability rather than physiological changes.^[8,21]^

GDM was diagnosed using the International Association of Diabetes and Pregnancy Study Groups/World Health Organization criteria, where one or more 75-gram OGTT values met or exceeded the following thresholds: fasting plasma glucose (FPG) ≥ 5.1 mmol/L, 1-hour plasma glucose (1h-PG) ≥ 10.0 mmol/L, or 2-hour plasma glucose (2h-PG) ≥ 8.5 mmol/L.^[1]^

Body mass index (BMI) was calculated as weight (kg) divided by height squared (m^2^) and categorized as underweight (<18.5 kg/m^2^); normal-weight (18.5–23.9 kg/m^2^); overweight (24–28 kg/m^2^); or obese (>28 kg/m^2^).^[22]^ Gestational weight gain (GWG) was defined as the difference between pre-delivery and preconception weight and classified as inadequate, adequate, or excessive based on the 2009 Institute of Medicine guidelines.^[23]^

HDP were defined as systolic blood pressure ≥ 140 mm Hg and/or diastolic blood pressure ≥ 90 mm Hg, measured on 2 occasions at least 4 hours apart, occurring at or after 20 weeks of gestation in a previously normotensive woman.^[24]^

### 2.3. OGTT

The 75-gram OGTT was performed between 24 and 28 weeks of gestation during routine prenatal visits. After an overnight fast, venous blood samples were collected at 0, 1, and 2 hours for plasma glucose measurements.

### 2.4. Clinical data and biochemical indicators

Demographic and clinical data, including maternal age, preconception weight, height, parity, gravidity, in vitro fertilization (IVF) status, GWG, gestational age, and comorbidities, were obtained from the hospital information system. Plasma glucose levels (FPG, 1h-PG, and 2h-PG) were measured using the hexokinase method on the Architect C16000 chemistry analyzer (Abbott, USA) in the hospital’s clinical laboratory.

### 2.5. Statistical analysis

Statistical analyses were performed using IBM SPSS 25.0 (Chicago, USA) for data analysis, and GraphPad Prism 9.0 (California, USA) was used to generate figures. Continuous variables were presented as mean ± standard deviation (SD), and categorical variables were expressed as frequencies and percentages [n (%)]. Independent-sample *t*-tests were used for comparisons between 2 groups, and chi-square tests were employed for categorical variables. Logistic regression analyses were conducted to calculate relative risks (RRs) and 95% CIs for HDP, with adjustments for covariates such as maternal age, preconception BMI, parity, gravidity, IVF, GWG, birth weight, gestational age, and GDM status. A significance level of *P* < .05 was considered statistically significant.

## 3. Results

### 3.1. Baseline characteristics stratified by glucose response curve

**Figure 2.**
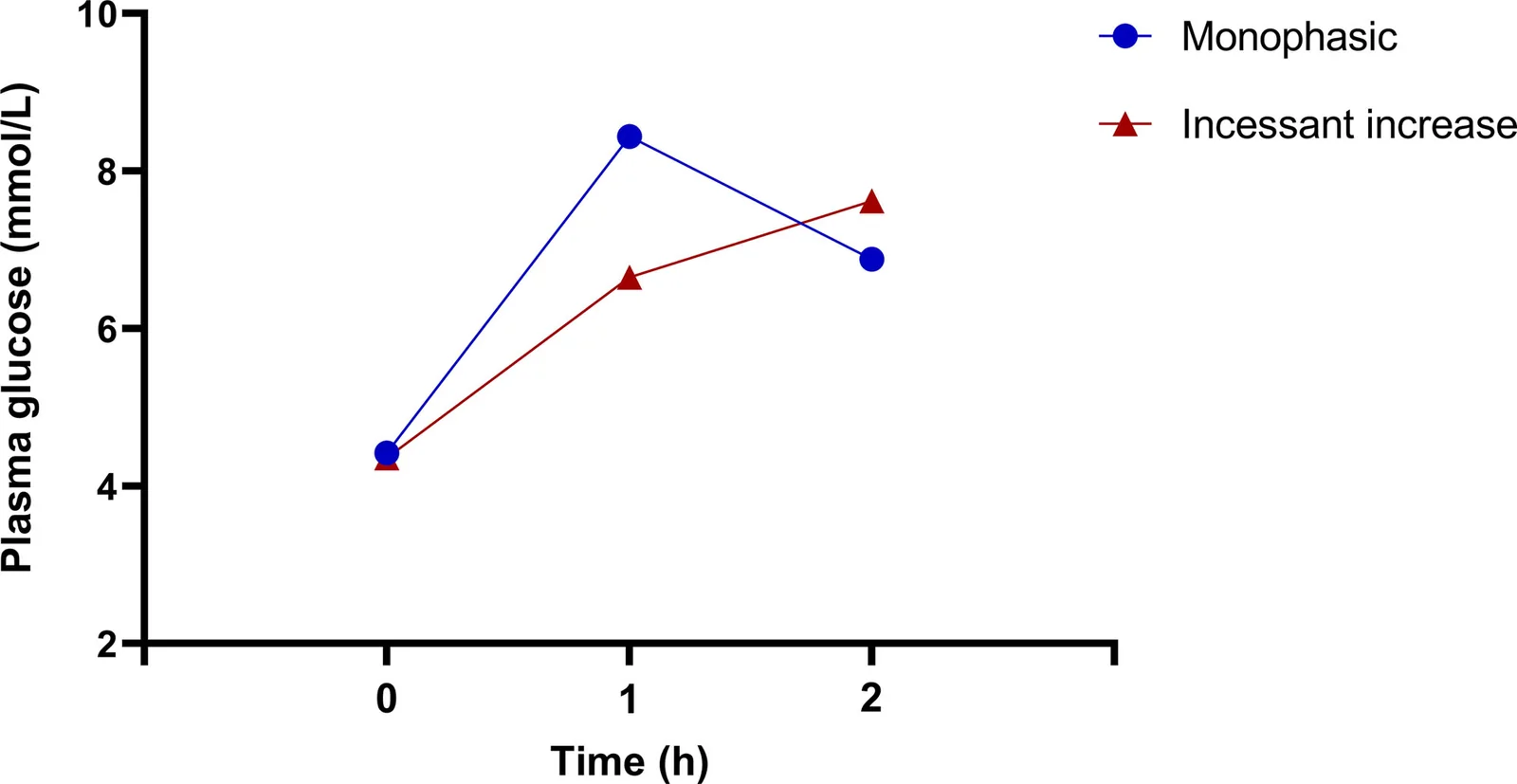
Plasma glucose concentrations during the OGTT in monophasic and incessant increase groups. OGTT = oral glucose tolerance test.

As presented in Table 1, 14.46% of the subjects exhibited an IIn pattern in the glucose response curve, while 85.54% showed a MPh curve. Figure 2 illustrates the average glucose levels during the OGTT for each group.

**Table 1:** Demographic characteristics of participants with incessant increase versus monophasic glucose response curves in OGTT.

Characteristics	All (n = 26,084)	Incessant increase (n = 3771, 14.46%)	Monophasic (n = 22,313, 85.54%)	*P*-value
Maternal age (yr)	31.2 ± 4.3	30.9 ± 4.2	31.2 ± 4.3	<.001
<35	20,339 (78.0)	3031 (80.4)	17,308 (77.6)	<.001
≥35	5745 (22.0)	740 (19.6)	5005 (22.4)	
Preconception BMI (kg/m^2^)	20.8 ± 2.7	20.6 ± 2.5	20.9 ± 2.7	<.001
Underweight	4852 (18.6)	779 (20.7)	4073 (18.3)	<.001
Normal-weight	18,075 (69.3)	2624 (69.6)	15,451 (69.2)	
Overweight and obese	3157 (12.1)	368 (9.8)	2789 (12.5)	
Parity (n, %)				.007
0	10,455 (40.1)	1599 (42.4)	8856 (39.7)	
1	7945 (30.5)	1098 (29.1)	6847 (30.7)	
≥2	7684 (29.5)	1074 (28.5)	6610 (29.6)	
Gravidity (n, %)				.003
Nullipara	15,792 (60.5)	2366 (62.7)	13,426 (60.2)	
Multipara	10,292 (39.5)	1405 (37.3)	8887 (39.8)	
IVF (n, %)	1186 (4.5)	147 (3.9)	1039 (4.7)	.039
GWG (kg)	13.9 ± 4.4	13.9 ± 4.4	13.9 ± 4.4	.753
Adequate	11,674 (44.8)	1656 (43.9)	10,018 (44.9)	.413
Inadequate	7038 (27.0)	1048 (27.8)	5990 (26.8)	
Excess	7372 (28.3)	1067 (28.3)	6305 (28.3)	
Birth weight (g)	3311 ± 446	3287 ± 449	3315 ± 446	<.001
Gestational age (wk)	39.1 ± 1.5	39.1 ± 1.6	39.1 ± 1.5	.223
OGTT (mmol/L)				
FPG	4.41 ± 0.37	4.36 ± 0.36	4.42 ± 0.37	<.001
1h-PG	8.18 ± 1.62	6.65 ± 1.49	8.44 ± 1.49	<.001
2h-PG	6.99 ± 1.42	7.62 ± 1.50	6.88 ± 1.38	<.001
GDM (n, %)	5313 (20.4)	892 (23. 7)	4421 (19.8)	<.001
HDP (n, %)	1300 (5.0)	140 (3.7)	1160 (5.2)	<.001

Continuous variables were presented as mean ± standard deviation and compared using the independent-sample *t*-test. Categorical variables were expressed as proportions (%) and compared using the chi-square test.

1h-PG = 1-hour plasma glucose, 2h-PG = 2-hour plasma glucose, BMI = body mass index, FPG = fasting plasma glucose, GDM = gestational diabetes mellitus, GWG = gestational weight gain, HDP = hypertensive disorders of pregnancy, IVF = in vitro fertilization, OGTT = oral glucose tolerance test.

A comparative analysis of the baseline characteristics between the IIn and MPh groups was conducted. The IIn group had significantly lower maternal age (*P* < .001) and BMI (*P* < .001). The rate of IVF was lower in the IIn group (*P* < .05), and neonatal weight was also significantly lower (*P *< .001). Regarding glucose-related parameters, the IIn group had higher 2h-PG levels (*P* < .001), but lower FPG (*P* < .001) and 1h-PG levels (*P* < .001). The incidence of GDM was significantly higher in the IIn group (*P* < .001).

Significant differences were also noted in parity and gravidity between the 2 groups (all *P* < .01). However, no significant differences were detected in GWG and gestational age (all *P* > .05). Notably, the prevalence of HDP was 3.70% in the IIn group, which was significantly lower than the 5.20% in the MPh group (*P* < .001).

### 3.2. Baseline characteristics stratified by HDP status

Table 2 shows the demographic characteristics of the participants, stratified by HDP status. The prevalence of HDP among the participants was 4.98% (1300/26,084).

**Table 2:** Demographic and clinical characteristics of participants stratified by HDP status.

Characteristics	HDP (n = 1300, 5.0%)	Non-HDP (n = 24,784, 95.0%)	*P*-value
Maternal age			<.001
< 35	945 (72.7)	19,394 (78.3)	
≥35	355 (27.3)	5390 (21.7)	
Preconception BMI (kg/m^2^)			<.001
Underweight	84 (6.5)	4768 (19.2)	
Normal-weight	770 (59.2)	17,305 (69.8)	
Overweight and obese	446 (34.3)	2711 (10.9)	
Parity			<.001
0	596 (45.8)	9859 (39.8)	
1	351 (27.0)	7594 (30.6)	
≥2	353 (27.2)	7331 (29.6)	
Gravidity			<.001
Nullipara	924 (71.1)	14,868 (60.0)	
Multipara	376 (28.9)	9916 (40.0)	
IVF (n, %)	126 (9.7)	1060 (4.3)	<.001
GWG			<.001
Adequate	465 (35.8)	11,209 (45.2)	
Inadequate	207 (15.9)	6831 (27.6)	
Excess	628 (48.3)	6744 (27.2)	
Birth weight (g)	3208 ± 590	3316 ± 437	<.001
Gestational age (weeks)	38.6 ± 1.8	39.1 ± 1.5	<.001
GDM (n, %)	369 (28.4)	4944 (19.9)	<.001
OGTT (mmol/L)			
FPG	4.55 ± 0.47	4.40 ± 0.36	<.001
1h-PG	8.65 ± 1.70	8.16 ± 1.61	<.001
2h-PG	7.26 ± 1.51	6.97 ± 1.42	<.001

Continuous variables were presented as mean ± standard deviation and compared using the independent-sample *t*-test. Categorical variables were expressed as proportions (%) and compared using the chi-square test.

1h-PG = 1-hour plasma glucose, 2h-PG = 2-hour plasma glucose, BMI = body mass index, FPG = fasting plasma glucose, GDM = gestational diabetes mellitus, GWG = gestational weight gain, HDP = hypertensive disorders of pregnancy, IVF = in vitro fertilization, OGTT = oral glucose tolerance test.

Compared with the non-HDP group, the HDP group had a higher proportion of advanced-aged mothers, overweight and obese individuals, along with a lower proportion of underweight individuals. The rate of IVF was higher in the HDP group. Moreover, the HDP group had a higher proportion of excessive GWG, lower neonatal weight, and a lower gestational age at delivery. The incidence of GDM was also higher in the HDP group.

There were significant differences in parity and gravidity between the 2 groups (all *P* < .001). In terms of glucose metabolism parameters, participants in the HDP group had significantly higher levels of FPG, 1-hour PG, and 2-hour PG compared to those without HDP (all *P* < .01).

### 3.3. RRs of HDP stratified by the glucose response curve

**Figure 3.**
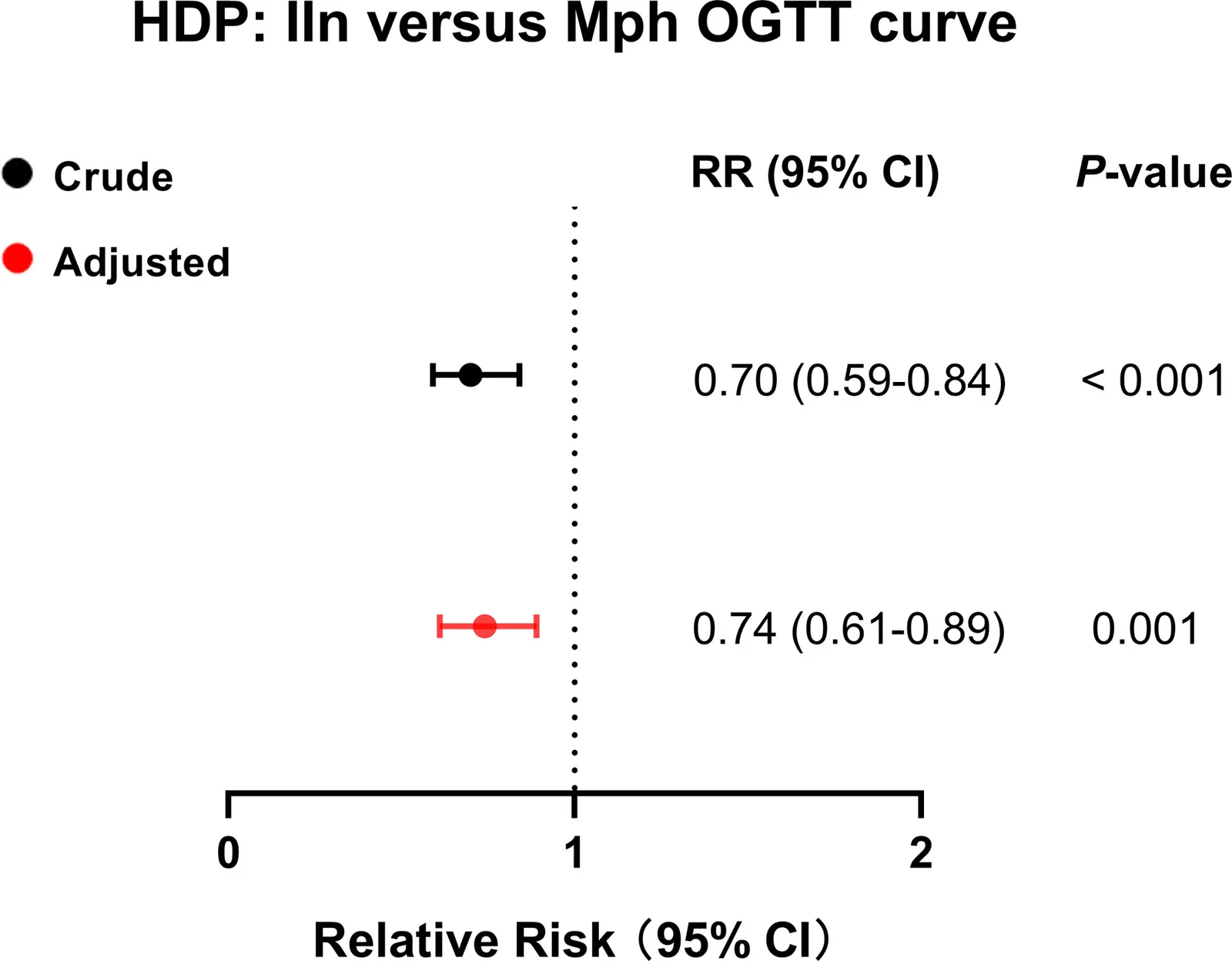
The relative risks of HDP among participants with monophasic or incessant increase curves. Crude, unadjusted; Adjusted, adjusted for maternal age, preconception body mass index, parity, gravidity, in vitro fertilization, gestational weight gain, birth weight, gestational age, gestational diabetes mellitus. HDP = hypertensive disorders of pregnancy, IIn = incessant increase, Mph = monophasic, OGTT = oral glucose tolerance test; RR, relative risk.

The risk of HDP was assessed among individuals with different glucose response curves, with those showing an MPh curve serving as the reference group. Logistic regression analysis was performed to quantify these risks (Fig. 3).

In the unadjusted (crude) model, prior to accounting for any confounding factors, individuals with an IIn curve had a lower risk of HDP compared to the MPh group. The relative risk and 95% CI were 0.70 (0.59–0.84) (*P* < .001).

Subsequently, in the adjusted model, where covariates such as maternal age, preconception BMI, parity, gravidity, IVF, GWG, birth weight, gestational age, and GDM status were taken into account, the IIn curve group still exhibited a reduced adjusted risk of HDP [adjusted RR: 0.74 (0.61–0.89), *P* < .01].

### 3.4. The relationship between 2 glucose response curves and HDP in women with varying demographic parameters

**Figure 4.**
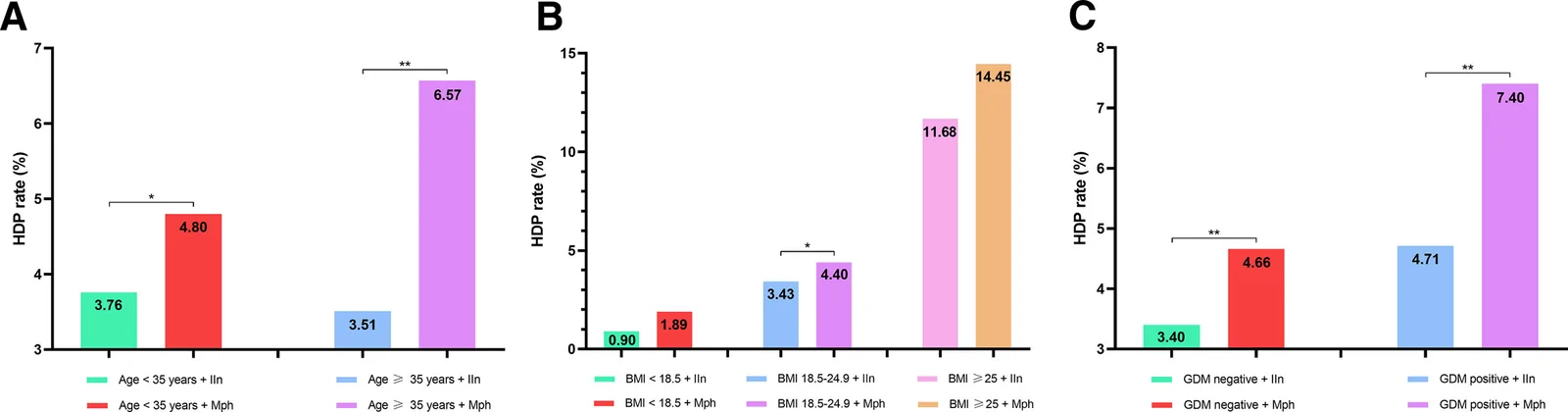
HDP rates in participants with different demographic parameters in monophasic and incessant increase groups. (A) HDP rates among 2 shapes of glucose response curve in different maternal ages; (B) HDP rates among 2 shapes of glucose response curve in different preconception BMI classification; (C) HDP rates among 2 shapes of glucose response curve in different GDM status. *P < .05; **P < .01. BMI = body mass index, GDM = gestational diabetes mellitus, HDP = hypertensive disorders of pregnancy, IIn = incessant increase, Mph = monophasic.

Maternal age, BMI, and GDM are widely recognized factors that are frequently linked to HDP. To explore the influence of these factors on the relationship between different glucose response curves and HDP, we categorized them. Maternal age was divided into 2 groups: those aged ≥ 35 years and those aged < 35 years. BMI was classified into 3 categories: normal-weight (BMI 18.5–24.9 kg/m^2^), underweight (BMI < 18.5 kg/m^2^), and overweight/obese (BMI ≥ 25 kg/m^2^). Regarding GDM, it was divided into GDM-positive and GDM-negative groups. Each of these groups was further divided according to the glucose response curve type, namely, IIn and MPh subgroups. As shown in Figure 4, numerically, all IIn subgroups had a lower incidence of HDP compared to their corresponding MPh subgroups.

### 3.5. RRs of HDP based on the glucose response curve in women with varying demographic parameters

**Figure 5.**
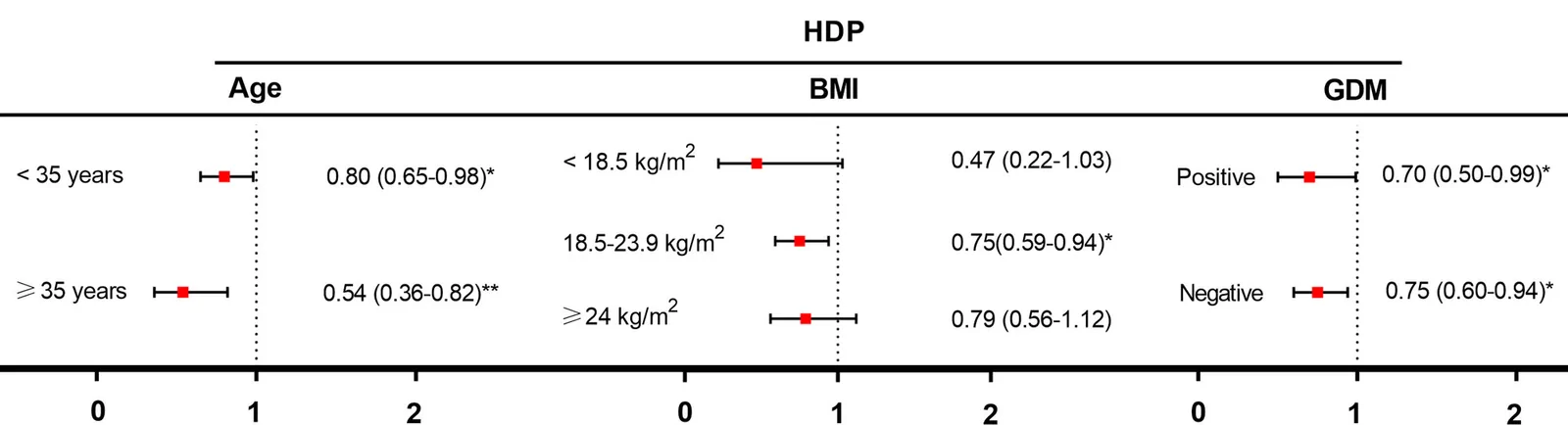
Relative risks of HDP in participants with monophasic and incessant increase curves by baseline characteristics. Relative risks were adjusted for maternal age, preconception body mass index, parity, gravidity, in vitro fertilization, gestational weight gain, birth weight, gestational age, gestational diabetes mellitus. *P < .05; **P < .01. BMI = body mass index, GDM = gestational diabetes mellitus, HDP = hypertensive disorders of pregnancy.

Subsequently, we evaluated the relative risk of HDP in the IIn group relative to the MPh group across different subgroups defined by demographic factors including maternal age, BMI and GDM status (Tables 3–5, Fig. 5).

**Table 3:** Relative risks of HDP based on the glucose response curve across different maternal age groups.

Subgroups	N (%)	Relative risks (95% CI)			
		Crude*	*P*-value	Adjusted†	*P*-value
Age < 35 yr	20,339				
Monophasic	17,308 (85.1)	Reference		Reference	
Incessant increase	3031 (14.9)	0.78 (0.64–0.95)	.012	0.80 (0.65–0.98)	.033
Age ≥ 35 yr	5745				
Monophasic	5005 (87.1)	Reference		Reference	
Incessant increase	740 (12.9)	0.52 (0.35–0.78)	.002	0.54 (0.36–0.82)	.004

Relative risks and 95% CIs were calculated using the logistic regression analyses and compared against the monophasic group.

CI = confidence interval, HDP = hypertensive disorders of pregnancy.

* Unadjusted.

† Adjusted for maternal age, preconception body mass index, parity, gravidity, in vitro fertilization, gestational weight gain, birth weight, gestational age, gestational diabetes mellitus.

**Table 4:** Relative risks of HDP based on the glucose response curve across different preconception BMI groups.

Subgroups	N (%)	Relative risks (95% CI)			
		Crude*	*P*-value	Adjusted†	*P*-value
Underweight	4852				
Monophasic	4073 (83.9)	Reference		Reference	
Incessant increase	779 (16.1)	0.47 (0.22–1.02)	.057	0.47 (0.22–1.03)	.060
Normal weight	18,075				
Monophasic	15,451 (83.0)	Reference		Reference	
Incessant increase	2624 (17.0)	0.77 (0.62–0.97)	.023	0.75 (0.59–0.94)	.011
Overweight and obese	3157				
Monophasic	2789 (86.8)	Reference		Reference	
Incessant increase	368 (13.2)	0.78 (0.56–1.10)	.153	0.79 (0.56–1.12)	.188

Relative risks and 95% CIs were calculated using the logistic regression analyses and compared against the monophasic group.

CI = confidence interval, HDP = hypertensive disorders of pregnancy.

* Unadjusted.

† Adjusted for maternal age, preconception body mass index, parity, gravidity, in vitro fertilization, gestational weight gain, birth weight, gestational age, gestational diabetes mellitus.

**Table 5:** Relative risks of HDP based on the glucose response curve across different GDM status groups.

Subgroups	N (%)	Relative risks (95% CI)			
		Crude*	*P*-value	Adjusted†	*P*-value
GDM-positive	5313				
Monophasic	4421 (83.2)	Reference		Reference	
Incessant increase	892 (16.8)	0.62 (0.45–0.86)	.004	0.70 (0.50–0.99)	.043
GDM-negative	20,771				
Monophasic	17,892 (86.1)	Reference		Reference	
Incessant increase	2879 (13.9)	0.72 (0.58–0.89)	.003	0.75 (0.60–0.94)	.011

Relative risks and 95% CIs were calculated using the logistic regression analyses and compared against the monophasic group.

CI = confidence interval, GDM = gestational diabetes mellitus, HDP = hypertensive disorders of pregnancy.

* Unadjusted.

† Adjusted for maternal age, preconception body mass index, parity, gravidity, in vitro fertilization, gestational weight gain, birth weight, gestational age, gestational diabetes mellitus.

Our findings demonstrated that, regardless of crude RRs or adjusted RRs, individuals in the IIn group had a significantly lower adjusted risk of HDP among both women aged < 35 years and those aged ≥ 35 years (Table 3) (all *P* < .05).

Moreover, in normal-weight women, regardless of whether the analysis was based on the crude model or the adjusted model, the IIn group exhibited a significantly lower risk of HDP, considering both crude RRs and adjusted RRs. In contrast, within the underweight and overweight/obese groups, no statistically significant differences were observed (Table 4) (all *P* > .05).

Finally, the results indicated that among individuals with or without GDM, in both the crude and adjusted models, those in the IIn group with GDM had a significantly reduced risk of HDP (Table 5).

## 4. Discussion

This study appears to be the first comprehensive report indicating that the IIn glucose response curves during OGTTs are independently associated with a reduced risk of HDP in Chinese pregnant women. In a cohort of 26,084 participants, 14.46% exhibited the IIn pattern. After adjusting for multiple potential confounders, including maternal age, preconception BMI, parity, gravidity, IVF, GWG, birth weight, gestational age, and GDM status, the IIn group still showed a significantly lower risk of HDP compared to the MPh group. This protective effect was also consistent across subgroups stratified by maternal age and GDM status, indicating the potential of the IIn pattern as a reliable biomarker for HDP risk stratification.

HDP has a profound impact on both maternal and fetal health, being influenced by a complex interaction of metabolic and physiological factors. Although numerous risk factors for HDP have been identified, a considerable number of HDP cases occur in women without apparent predisposing conditions. Hyperglycemia has been established as an independent risk factor for HDP.^[25]^ However, previous studies mainly concentrated on whether the 3 blood glucose values obtained from OGTTs were elevated, often neglecting the overall OGTT response curve. To date, only a few investigations have focused on incessant increasing glucose response curves. Most previous research classified glucose response curves during OGTTs into monophasic, biphasic, or triphasic patterns and explored their associations with other adverse outcomes. Unfortunately, some studies excluded participants with incessant increasing curves, categorizing them as having an “anomalous”^[26]^ or “unclassified” shape.^[16,20]^ As a result, there is limited knowledge regarding the distribution characteristics and clinical significance of populations with an IIn curve. Several studies have explored the physiological mechanisms underlying the IIn glucose response curve. The IIn pattern is characterized by a “delayed” glucose response. In our study, the fasting blood glucose in the IIn group (4.36 ± 0.36 mmol/L) was slightly lower than that in the MPh group (4.42 ± 0.37 mmol/L). Other studies have shown that the insulin responses to glucose did not differ between the IIn curve and MPh subgroups, while the IIn subgroup had lower overall insulin levels.^[6]^ Given that strong evidence suggests an association between insulin elevation and hypertension,^[13]^ investigating the relationship between the IIn group, which is associated with low insulin levels, and the HDP group, which is associated with high insulin levels, is an interesting area of research.

Given the potential link between the IIn glucose response curve and HDP, and the scarcity of research on this relationship, our study aimed to fill this gap. We investigated the correlation between different OGTT glucose response patterns and HDP risk in a large cohort of Chinese pregnant women. Our results revealed that the IIn pattern was associated with a significantly lower HDP risk compared to the MPh pattern. This protective effect persisted after adjusting for confounding factors. Studies have demonstrated that pregnancy is characterized by complex hormonal changes that induce gestational IR and elevated insulin levels, which were linked to the metabolic effects of progesterone, human placental lactogen, free cortisol, and estrogen in the maternal circulation.^[27]^ IR refers to reduced tissue sensitivity to insulin, impairing its ability to promote glucose uptake and utilization. To compensate, pancreatic β-cells enhance insulin secretion, resulting in hyperinsulinemia – a key compensatory mechanism in response to IR.^[28]^ In physiological pregnancy, reduced insulin sensitivity increases glucose output and decreases glucose uptake/utilization, ensuring sufficient energy supply to the fetus.^[29]^ Most healthy pregnant women can counteract peripheral IR by markedly increasing basal and nutrient-stimulated insulin secretion. However, insufficient β-cell compensation leads to hyperglycemia, ultimately progressing to GDM.^[30]^ Insulin resistance and hyperinsulinemia are considered common pathological underpinnings of hypertension and diabetes.^[27]^ The core pathogenesis of HDP involves vascular endothelial dysfunction and systemic vasospasm, with insulin resistance and hyperinsulinemia acting as critical drivers. Specifically, insulin resistance promotes HDP development by damaging vascular endothelium, activating the renin-angiotensin-aldosterone system, overstimulating the sympathetic nervous system, and amplifying inflammatory and oxidative stress responses.^[31]^ Notably, despite a significantly higher incidence of GDM in the IIn group compared to the MPh group (23.7% vs 19.8%, *P *< .001) – and GDM being a known risk factor for HDP^[32]^ – the IIn group still exhibited a lower HDP risk (3.7% vs 5.2%, *P *< .001). This paradoxical observation stems from the unique metabolic features of the IIn curve. The higher GDM rate in the IIn group is primarily driven by sustained elevation of 2h-PG, resulting from impaired insulin secretion. This delayed insulin response impairs late-phase glucose clearance during OGTT, increasing GDM diagnosis. Thus, even with lower FPG and 1h-PG, the isolated elevation of 2h-PG in the IIn group suffices to meet GDM diagnostic criteria, explaining its higher GDM incidence.

In contrast, HDP pathogenesis is closely linked to hyperinsulinemia and its downstream effects on vascular function. Compared to the MPh group, individuals with the IIn curve exhibit reduced insulin secretion.^[6,7]^ Excess insulin (as seen in the MPh group) promotes endothelial dysfunction by inducing oxidative stress and reducing nitric oxide production, activates renin-angiotensin-aldosterone system to enhance vasoconstriction, and increases sympathetic nervous system activity and sodium retention – all key drivers of HDP. However, in the IIn group, lower insulin levels may mitigate these processes, counteracting the HDP-promoting effects of higher insulin. Moreover, the temporal pattern of glucose elevation in IIn curves (a continuous rise rather than an early peak) may differentially affect vascular physiology. Unlike the MPh pattern, where acute glucose spikes trigger rapid insulin surges, the gradual glucose increase in IIn curves may reduce oxidative stress and inflammatory responses in vascular endothelium – pathways that, when overactivated, contribute to gestational hypertension. This may explain why the IIn pattern confers protection against HDP despite GDM-related hyperglycemia. Consistent with our findings, Zhang et al^[7]^ reported a significantly lower prevalence of hypertension in the IIn group compared to the MPh group among patients with type 2 diabetes, validating the reliability of our results.

Advanced maternal age, elevated BMI values, and the presence of GDM status are widely recognized as established risk factors for HDP.^[33–35]^ Our research data showed a statistically significant difference between the IIn curve and MPh groups. However, due to the scarcity of studies examining the relationship between OGTT curves and HDP in combination with these critical risk factors, we conducted a stratified analysis based on maternal age, preconception BMI, and GDM status. Compared with the MPh group, all the IIn curve subgroups had a lower risk of HDP. The IIn group was a protective factor for HDP in both elderly and normal-aged pregnant women, as well as regardless of GDM. In the normal-weight category, the IIn subgroups also had a lower risk of HDP. However, no significant differences were observed in the underweight or overweight/obese subgroups.

This study has several strengths. First, in contrast to previous studies that mainly focused on non-pregnant populations or different OGTT patterns in nonspecific groups, our study specifically investigated the distribution of OGTT curve shapes and their clinical implications in Chinese pregnant women. It provided novel evidence of a potential association between the IIn glucose response curve and a reduced risk of HDP, which had not been explored in this population before. Second, our research findings offer clear, evidence-based insights for obstetricians, who often face confusion and uncertainty regarding the impact of continuously elevated OGTT curves on pregnancy outcomes, thus improving clinical decision-making and patient care. Third, the IIn pattern demonstrated a consistent protective effect across subgroups stratified by maternal age and GDM status, suggesting its robustness as a potential biomarker for HDP risk stratification. Fourth, our study benefited from a large sample size of 26,084 participants, which enhances the reliability and generalizability of the findings.

Nevertheless, this study has limitations. First, the study population was restricted to Chinese pregnant women, and the generalizability of the findings to other ethnic groups remains to be determined. Future research should validate these findings in diverse populations. Second, due to the retrospective nature of the study, we were unable to collect data on lifestyle interventions, treatments received by the participants, or their therapeutic efficacy, which could have provided additional insights. Third, the lack of information on insulin or C-peptide, parameters not routinely assessed in clinical practice, limits our ability to fully understand the underlying mechanisms linking OGTT patterns to HDP. Fourth, certain confounders, such as special diets, genetic factors, and metabolic status, and other lifestyle factors, could not be collected, potentially influencing the observed outcomes. Finally, while our study identified the IIn pattern as a protective factor, the proportion of individuals exhibiting this curve was relatively small compared to the monophasic group. Therefore, additional research involving larger and more diverse populations is essential to validate these findings. Moreover, in-depth mechanistic investigations are required to elucidate the biological pathways that link glucose response patterns, particularly the IIn pattern, to the risk of HDP.

## 5. Conclusion

This study reveals a significant association between the glucose response curve during OGTT and the risk of HDP, with the IIn pattern acting as a protective factor. The consistent protective effect of the IIn pattern across demographic subgroups highlights its potential utility in identifying low-risk individuals and guiding personalized management of pregnancy-related complications. Although the proportion of individuals with the IIn curve is smaller than that of the monophasic pattern, its clinical significance merits additional attention from clinicians and researchers to further explore its implications. These findings contribute to a more profound understanding of the intricate interplay between glucose metabolism and HDP, paving the way for targeted interventions to enhance maternal and fetal outcomes. Future research should focus on exploring the underlying mechanisms linking glucose response patterns to HDP risk and validating these findings in diverse populations to improve their generalizability.

## Author contributions

**Conceptualization:** Min Guo, Ya Xi, Yongying Bai.

**Data curation:** Jinghua Zhang.

**Methodology:** Kaiqi Wu.

**Software:** Huiqing Yang, Binbin Yin.

**Supervision:** Yongying Bai.

**Writing – original draft:** Min Guo, Kaiqi Wu.

**Writing – review & editing:** Ya Xi, Yongying Bai.
